# Improving medical students’ communication competencies to deal with intimate partner violence using clinical simulations in Mozambique

**DOI:** 10.1186/s12909-021-02560-8

**Published:** 2021-02-23

**Authors:** B. Manuel, M. Valcke, I. Keygnaert, K. Roelens

**Affiliations:** 1 Department of Community Health, Faculty of Medicine, University Eduardo Mondlane, Av. Salvador Allende, nr 702, Maputo, Mozambique; 2 Department of Educational Studies, Ghent University, Ghent, Belgium; 3 Department of Public Health and Primary Care, International Centre for Reproductive Health, Faculty of Medicine and Health Sciences, Ghent University, Ghent, Belgium; 4 Department of Obstetrics and Gynaecology, Faculty of Medicine and Health Sciences, Ghent University, Ghent University Hospital, Ghent, Belgium

**Keywords:** Communication competency, Medical education, Intimate partner violence, Undergraduate, Physician-patient communication, Pre-post-test, Clinical simulation, Role plays

## Abstract

**Background:**

During their medical training, medical students aim to master communication skills and professionalism competencies to foster the best possible patient-physician relationship. This is especially evident when dealing with sensitive topics. This study describes and analyses the outcomes of a simulation-based training module on clinical communication competency through interacting with simulated intimate partner violence (IPV) survivors. The training was set up as part of a broader IPV module within a Gynaecology and Obstetrics Bachelor of Medicine and Bachelor of Surgery of Medicine (MBBS).

**Methods:**

In total, 34 (59%) of all fourth-year medical students from one medical school in Mozambique were involved. A mixed-method approach was adopted. First, a quasi-experimental pre-test/post-test design was adopted to study the impact of the intervention to tackle critical IPV knowledge, skills, and attitudes, underlying a patient communication script. Second, a qualitative analysis of student perceptions was carried out.

**Results:**

The results of the paired sample t-tests point at a significant and positive change in post-test values when looking at the general IPV self-efficacy (IPV SE) score and the subscales mainly in attitudes. Participants expressed a desire for additional IPV communication competency and suggested enhancements to the module.

**Conclusion:**

We conclude that due to IPV being a sensitive issue, simulation activities are a good method to be used in a safe environment to develop clinical skills. The results of this study are a good complement of the analysis of the competencies learned by the medical students in Mozambique with the current curriculum.

**Supplementary Information:**

The online version contains supplementary material available at 10.1186/s12909-021-02560-8.

## Background

Mastery of communication competency is critical for medical students to assist patients. Adequate communication competencies promote physician-patient communication and the development of a proper professional relationship with the patient. Medical schools are starting to recognize the importance of these competencies in their curriculum [1].

Though a variety of concepts is used in the literature – interpersonal communication, clinical communication – we adopt in the present article the key concept of communication competency. It is defined as “face to face verbal or non-verbal exchange of information and feelings between two or more people” [2]. Effective communication between healthcare providers and patients is vital for the improvement of client satisfaction, compliance, and health outcomes. Patients who understand the nature of their illness and its treatment, and who believe the provider is concerned about their well-being, show greater satisfaction with the care received and are more likely to comply with treatment regimens [3].

Mastery of communication competencies enables tackling complex topics that are less comfortable for the patient. Intimate partner violence (IPV) is considered a complex topic and many physicians avoid addressing it [4–6]. It is sensitive for both physicians and survivors with challenges occurring mainly during screening and referrals. Some physicians feel overwhelmed by information provided by survivors regarding their abusive relationships, especially when they do not know how to respond or who to refer them to. Victims may also feel uncomfortable sharing information about their relationships and be reluctant to disclose IPV [7–9].

Clinical simulations combining standardized actors, role play, simulated patients, group discussions, and debriefing have been highlighted to develop communication competencies [1]. Training on the base of a variety of clinical simulation scenarios that medical students may encounter in the future can help to deal with discomfort in both victims and future physicians [10–13] The literature also points at background variables that might influence physicians’ IPV competencies, such as sex, sexual orientation, marital status (married, single, cohabiting, divorced, and widow), number of IPV survivors assisted, and whether the students themselves have experienced IPV or witnessed IPV in their own family.

In general, little information is known about medical students’ comprehensive mastery of IPV curriculum contents and how this sensitive topic should be taught to acquire better physician-patient communication skills. At the time of this study, no formal communication skills training related to IPV exists in the Mozambique setting for medical students [14]. In the present study, a module was designed to train medical students to acquire communication competencies in medical students in Mozambique when dealing with IPV survivors during a consultation session. These specific communication competencies build on a set of knowledge, skills, and attitudes concerning IPV patients, their families, the context, and also with a medical physician’s concerns (as being also a victim, sharing the same cultural points of view of the victims, and little or no training on dealing with IPV) [13]. We mainly aim at developing a foundation in the acquisition of IPV-related knowledge, skills, and attitudes in the context of a patient consultation session. Also, the clinical experiences are expected to boost students’ self-efficacy in their IPV communication competency. In the medical education literature, self-efficacy is accepted as a precursor of future behaviour and performance [15, 16].

## Methods

A mixed-methods approach was adopted, consisting of a quasi-experimental pre-test/post-test design without comparison groups, and a qualitative analysis of students’ perceptions about the IPV module.

Ethical approval was obtained from the Institutional Committee of Bioethics and Health linked to the Faculty of Medicine/Central Hospital of Maputo, Mozambique (registration number CIBS FM & MCH/006/2016). Also, permission was granted by the dean of the medical school and the lecturer in gynaecology and obstetrics.

### Study location

The training was implemented from April to May 2019, in meeting rooms of the General Hospital of Mavalane, Central Hospital of Maputo and classrooms of the Community Health Department of the Faculty of Medicine of the Eduardo Mondlane University, all in Maputo city, Mozambique.

### Participants

Participants in this study were volunteering medical students, enrolled for the fourth year in the gynaecology and obstetrics rotation in the Faculty of Medicine at the Eduardo Mondlane University, Mozambique. We contacted the lecturer in gynaecology of the fourth-year rotation at the Faculty of Medicine from the Eduardo Mondlane University telephonically regarding the study and she provided the phone number of the student representative. The student representative was the one in charge of contacting all her classmates in the rotations and organizing dates and times of the training in the different locations. From the 58 students enrolled in the gynaecology rotation, 59% participated. All 34 students who volunteered were considered eligible, and their data were included in the analysis. Their average age was 24 years and 6 months; 20 were female, 27 described their sexual orientation as heterosexual, 24 were single, 7 reported being survivors of IPV themselves and 13 witnessed IPV in their family (see Table 1). These background variables were considered when studying the potential impact of the clinical simulation.

**Table 1:** Socio-demographic characteristics of study participants

Socio-demographic characteristics	n	%
**Age, years**		
20 to 25 years old	21	61,8
≥ 25 years old	13	38,2
Mean age	24,64	
**Sex**		
Female	20	64,5
Male	11	35,5
**Sexual orientation**		
Heterosexual	27	87,1
Men who have sex with men (MSM)	1	3,2
Bisexual	1	3,2
**Marital status**		
Married	3	9,7
Single	24	77,4
Cohabiting	3	9,7
**Survivor of IPV**		
Yes	13	41,9
No	16	51,9
**Survivor of IPV by sex**		
Female	6	19,35%
Male	1	3,22%

All participants signed an informed consent form after receiving an explanation about the focus of the training, confidential treatment of collected data, the voluntary nature of participation, and their right to withdraw at any time. Random numbers were given to participants to pair questionnaire responses to pre- and post-intervention instruments.

### Structure and validity of the clinical simulation-based training

A new training module was designed with the aim of developing the communication competency to deal with IPV survivors. This implied three key design and development activities. First, real-life cases – focusing on communicating with IPV survivors in a consultation setting – were collected to guide the build-up of the clinical simulations. The collection was influenced by WHO and local sources. Expert opinions were used to validate the cases in the specific Mozambique setting. Scenarios were developed for each case in view of the clinical simulations. Second, the communication competency was operationalized by the development of a script that serves as a protocol when communicating with IPV survivors. This script included knowledge, skills, and attitude components considered critical when dealing with IPV. It was developed and validated in the context of an earlier study [13]. Next, instruments were designed to measure the impact of the intervention on students’ self-efficacy related to the IPV communication competency as reflected in their IPV-related knowledge, skills, and attitudes.

### Design of the clinical simulations: case and scenarios

Each scenario – duration between 2 and 3 min – focused on the physicians’ interaction during a different real-life clinical situation. The case scenarios were screened by a multidisciplinary team of physicians (*n* = 3), psychologists (*n* = 4), and forensic physicians (*n* = 3) working at the Integrated Care Centre for Victims of Violence from the General Hospital of Mavalane and the Forensic Medicine Department of the Central Hospital of Mozambique, in Maputo, Mozambique, to evaluate their quality in terms of content, adequacy, and realism. We had a follow-up meeting after a previous visit that we made to the centre and to the Central Hospital to understand the workflow of both. In that meeting, we invited the multidisciplinary team to comment on the scenarios. In total, we developed three case scenarios to guide clinical simulations. Any discrepancies in the evaluation of scenarios were resolved by consensus, building on the experience of the multidisciplinary team.

For each specific scenario, a written guide was developed [17]. In this guide, we first described the context (see Additional file 2). Next, we described different roles: physician, survivor, an accompanying family/community member, and an assailant or alleged assailant. We offered a brief about each role, a description of scenes that would take place, and a necessary role-specific dialogue allowing for a certain level of personal interpretation.

### Design of a script to guide the IPV communication competency

IPV-related knowledge, skills, and attitudes interact in IPV communication competencies and play a role throughout a script, directing a specialized patient consultation when dealing with IPV. This script can be seen as a protocol to be followed by students in a flexible way. We designed a script on the base of the Government of Canada’s “Talking Tools 1 and 2” [18, 19] combined with modules on IPV from medical schools in Mozambique, NGOs, and the Ministry of Health of Mozambique, and a literature review submitted to a peer-review journal conducted by the researchers. This script is available online [20].

### Research instruments

First, a background questionnaire was developed to collect information about students’ age, sex, sexual orientation, marital status, and whether they had personally experienced IPV. Some additional questions focused on whether they had experienced IPV in their family, and whether they had already received IPV-related training. This background information was considered when developing the case scenarios. Second, a self-efficacy scale was developed, following Bandura’s guidelines [21]. The scale consisted of 21 items mapping knowledge, skills, and attitudes that play a role in the communication competency. The students indicated the “strength of their efficacy beliefs on a 100-point scale, ranging in 10-unit intervals from 0 (“Cannot do”); through intermediate degrees of assurance, 50 (“Moderately certain I can do”) to complete assurance, 100 (“Highly certain I can do”)” [21]. This instrument was developed and validated in an earlier study [13] and is available in Additional file 1. Sample item: “( …) Understanding barriers to an abused person admitted that s/he was abused and seeking help” and “Being able to develop a strategy for interacting with a local community when an IPV case is identified (such as police, social services).” The internal consistency – calculated with Cronbach’s Alpha – ranges in the present study from 0.83 (pre-test) to 0.93 (post-test). Thirdly, in view of the qualitative part of the study, six open-ended questions were presented, each on a separate-coloured card: (1) What was most challenging in this training? (2) What was the most remarkable part of the training? (3) What was positive? (4) What was negative? (5) What do you think should improve on as a physician? (6) What did you learn? [22].

### Research procedure

**Fig. 1:**
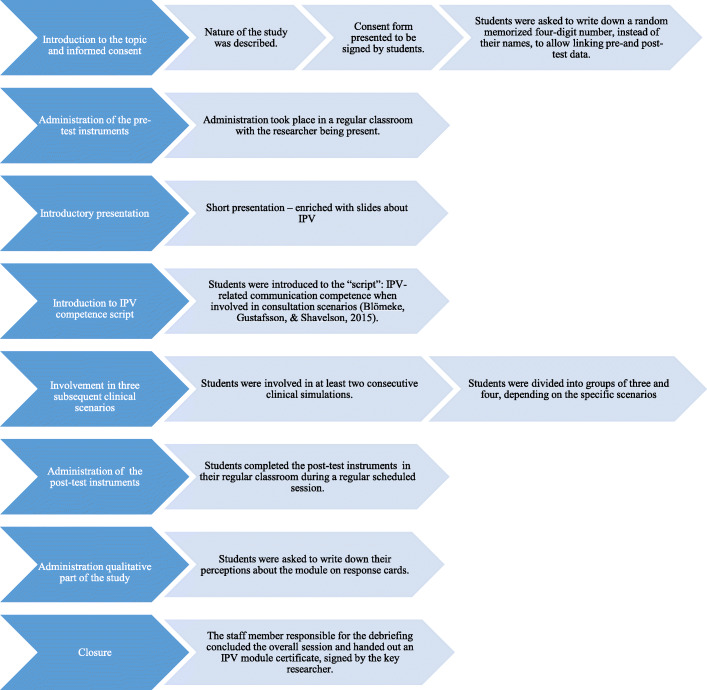
Flow diagram depicting the procedure in the study

Figure 1 depicts the procedure of the study. The students played the roles of medical physicians, patients, and accompanying family and observers in the clinical simulations based on the provided scenarios. The number of students in each group ranged from 7 to 16. All the students were actively involved in at least two case scenarios. The qualitative part of the study in which students responded to the six open-ended questions also functioned as a debriefing session.

### Quantitative statistical analyses

Statistical analysis was carried out using IBM® SPSS® Statistics 25.0 and the level of significance adopted for all tests was *p* < 0.05.

Data analysis started by calculating descriptives (see Table 2). Next, sum scores were calculated for the complete IPV SE scale and the IPV SE subscales focusing on the (a) knowledge, (b) skill, and (c) attitude elements in the script. Internal consistency of these scales was evaluated for both pre- and post-test administration using Cronbach’s Alpha (α), with the following benchmarks: poor when α < 0.6, acceptable when between 0.6 and 0.8, and good when α ≥ 0.8 [23]. Data normality was verified using the Kolmogorov–Smirnov test.

**Table 2:** Summarizes the descriptives of the research variables M (SD)

Variable	N	Mean (SD)
Pre-test IPV SE Knowledge	34	47,38 (19,11)
Post-test IPV SE Knowledge	34	78,66 (19,86)
Pre-test IPV SE Skills	34	59,07 (20,21)
Post-test IPV SE Skills	33	79,72 (18,80)
Pre-test IPV SE Attitudes	34	45,09 (24,92)
Post-test IPV SE Attitudes	33	76,44 (18,18)

A paired sample t-test was applied to assess the impact of the clinical simulations on changes in students’ IPV self-efficacy from pre- to post-test. Next, difference scores were calculated (post-test minus pre-test) for the total self-efficacy scale and the subscale scores. To check the impact of background variables, ANOVA was carried out – using the difference scores – with background variables as factors: age groups (≤25 years or > 25 years), sex (male or female), sexual orientation (heterosexual, MSM, bisexual), and whether they were a survivor of IPV (yes/no).

### Qualitative statistical analyses

One researcher coded the data, obtained through the responses on the six coloured cards. Content analysis was applied to identify themes and subthemes when screening the answers to each of the six questions [24].

## Results

### Changes in students’ IPV SE

The results of the paired sample t-tests point at a significant and positive change in post-test values when looking at the general IPV SE score and the subscales: IPV SE Knowledge (*t* (31.28) = 7.27, *p* < .01); IPV SE Skills (*t* (19.46) = 5.60, *p* < .01); IPV SE Attitudes (*t* (30.28) = 5.97, *p* < .01); general IPV SE (*t* (28.19 = 7.67, *p* < .01).

### Association with background variables

None of the background variables appear to play a role. The related analysis of variance results is presented in Table 3.

**Table 3:** Analysis of variance test results, building on background variables as factors

	General IPV SE	SE IPV–Knowledge	SE IPV–Skills	SE IPV–Attitudes
	*F*	*F*	*F*	*F*
Age	1,38	1,63	0,23	0,78
Sex	0,05	0,17	0,19	0,43
Sexual orientation	0,43	0,05	0,68	0,98
Marital status	1,84	1,95	1,23	0,73
IPV survivor	0,04	0,31	0,27	1,15

*p < 0,05

### Student perceptions: a qualitative analysis

As explained earlier, students received during a debrief six cards, each with a specific question.

The students’ reflection for each question was analysed to identify key themes and subthemes. We report below the themes that occurred the most and/or themes reflecting conflicting opinions. At the same time, the reflections also help understand the positive impact of the intervention on students’ IPV self-efficacy.

#### Understanding the positive impact of the intervention on students’ IPV SE

Students reported that the training helped to develop a broader view of what IPV is. Especially their misconception that IPV only appeared as physical violence was corrected. They also reported that the training helped them in how to address the topic with patients and the how-to guide questions for interviews.

#### Safety

The students reported that the clinical simulation training was a safe environment to help them focus on their feelings and accept feelings of each other. For example, *the simulated consultations showed me some aspects of the day-to-day in the wards and consultations that I unconsciously do wrong,* e.g.*, attention to eye contact, the issue of empathy and others.*

#### IPV competencies

Students’ IPV SE clearly reflected the multidimensional nature of IPV as a construct. Regarding knowledge, students reported that after the training, they could define IPV, and recognize the signals and symptoms, or dynamics. The students reported that with the clinical simulations, they were also able to understand the magnitude, prevalence, and impact of direct and indirect exposure to IPV. For example, the training *(…) was an illustration of the great importance it has for the undergraduate Medicine training, a subject or module that teaches students and future physicians, how to deal with a patient suffering from IPV.*

Regarding skills, the students reported that they had learned how to address the topic with survivors in an empathic, non-judgmental manner. For example, *it is necessary to have posture and empathy and respect for the victim so that the victim collaborates in every way, and we can provide support.*

The students reported strong feelings related to overcoming own stereotypes surrounding IPV. They also reported that they had learned how to overcome the common barriers for a physician to identify a survivor of IPV and learned how to conduct a detailed history when IPV is suspected. For example, *it was challenging to leave aside my personal views about the relationship between people from the same sex.*

The students also reported that the training had helped in understanding the limits of confidentiality surrounding the discussion of IPV and overcoming stereotypes. For example, *the most remarkable part of the training was the case of sexual assault perpetrated by an intimate partner in a man who have sex with men (MSM) couple.*

#### Curriculum

Related to time and curriculum it was reported that this type of training should be introduced in the medical curriculum to make communication competency learning interactive and standardized. However, the students reported that they needed more time to complete the training. For example, *it should be given at least 1 week*, *we have not had enough time to debate over the issue, clarify doubts and do more exercises.* The students also reported that *I would also like to make it clear that a treatment to approach abusers is also important.*

## Discussion

We conducted this study to understand whether there is a difference in students’ SE in IPV-related communication competencies before and after being involved in clinical simulations. Simulation has been described as a “more effective method in changing behaviours and thought processes” [11]. It can provide “a secure and protected learning environment for students to develop clinical skills without the risk of patient harm” [11]. Besides, we checked whether students’ backgrounds influence this expected change in SE. Our module was scheduled during the gynaecology rotation of the fourth-year’ students to minimize the burden of time by allowing it to fit into already existing schedules. However, we had 59% participation. Overall, the participants believed that the training they received was valuable. The results of the paired sample t-tests point at a significant and positive change in post-test values when looking at the general SE score and the subscales and mainly in attitudes. Participants also expressed a desire for additional education and suggested enhancements to the module. To consider the impact of background variables, difference scores in changes in self-efficacy were compared for the different age groups, sex, and personal IPV experience. Analysis of variance only points at significant differences in older students. The self-efficacy perception of the students on their learning can be considered a reasonable evaluative parameter, since the self-efficacy has been correlated to the practice of communication skills and has shown to improve their outcomes [1, 21, 25]. However, it is also significant in young students as it is crucial that this training can also affect them as the literature recommends that the students have contact with the subject at the early stages of their medical curriculum [13]. Students reported that the training helped to have a broader view of what IPV entails because they assumed it only regarded physical violence. They also reported that the training helped them in how to address the topic with patients with the help of the how-to guide questions for interviews, how to deal with the referral points, and the impact and national dimension of IPV.

Regarding the positive impact related to curriculum methodology, students reported that playing a role helped to understand better the attitudes that a physician should adopt, and that the training was very interactive. Students also suggested that this topic could be part of the curriculum of healthcare providers and they recognized that they need to be trained in this specific area for better care of their future patients, in their family and/or community. Practicing communication skills in a physician’s role and scripts of patient roles mimicked well the reality that the students may face in practice as already reported in previous studies [10–12]. Learning was based on students’ active observations and their reflections about communication competencies shown in the role plays [26]. This type of teaching and learning is new in the Mozambique setting [14] and is an exemplar for tackling (transferring) other critical competencies (psychiatry, internal medicine, paediatrics, and so on). This module should involve more than just a single training session. It should be an ongoing initiative to ensure that participants continue to retain and enhance their knowledge gains. Participants expressed plans to incorporate training into their practice. The module must be addressed transversally and should/could be made to IPV problems in other domains.

## Limitations

This study has limitations, which must be taken into consideration when evaluating and interpreting the results. The Hawthorne effect may have impacted the results since the medical students knew that their performance during the training was being observed for analysis and publication. It is unclear whether a detailed intervention would be more or less effective among students who may have received more exposure to IPV training. The findings of medical students’ communication competencies in these studies may be representative of this cohort, but not necessarily of all medical students. We triangulated the quantitative data with qualitative data of the module. The small number of the sample could limit its power to identify relevant factors. Other than that, the group’s variability, the short duration of the module, and the results of the assessment of the module taken solely after its completion are the main limiting aspects of the study. As all the participants were volunteers, it is possible that the participants were motivated students. The pre-test/post-test design in these studies means that differences found in self-reported learning outcomes may be due to factors other than the module. A randomized controlled trial should have been designed to understand the direct impact of the module better. The size of the groups varied between seven and 17 participants, and this could have possibly influenced the learning stimulus and the results of the study. To further evaluate the effectiveness of the module, we could have used observation combining practical sessions in clinical settings. It has been suggested that communication training should be integrated with clinical experience and an actual healthcare visit may help medical students to appreciate communication skills at an early stage of their studies.

As this is an uncomfortable topic, and the students can also be victims of IPV, an online module could be used to avoid discomfort with the subject. Further studies must provide an analysis of results in larger samples. Professional secrecy and how to go about IPV when, for example, the physician feels the patient needs more help from other healthcare professionals or police is an important debate at the moment in IPV and should also be a topic for further studies. Apart from that, a follow-up test can be useful to evaluate the content fixation. We plan to do a follow-up questionnaire. This is the first module and the first study conducted in Mozambique on communication competencies in IPV. The training needs to be systematically integrated into the whole of the curriculum of medical schools in Mozambique.

## Conclusion

With the results of this IPV SE training, we concluded that IPV being a sensitive issue, simulation activities are a good method to be used in a safe environment to develop clinical skills. We also concluded that students reported that they felt more competent after participating in this training in a safe environment. The results of this study are a good complement of the analysis of the competencies learned by the medical students in Mozambique with the current curriculum [13].

## Supplementary Information

**Additional file 1.**

**Additional file 2.**

## Data Availability

The datasets used and/or analysed during the current study are available from the corresponding author on reasonable request.

## Abbreviations

IPV: Intimate partner violence
IPV SE: Intimate partner violence self-efficacy scale
MSM: Men who have sex with men
