# Supplementary material for: Improving medical students’ communication competencies to deal with intimate partner violence using clinical simulations in Mozambique
Source: BMC Med Educ. 2021 Feb 23;21:126. doi: 10.1186/s12909-021-02560-8 (PMC7901116; doi:10.1186/s12909-021-02560-8)
Supplement: Supplementary file 1 — Additional file 1. [file 12909_2021_2560_MOESM1_ESM.docx]

**Additional file 1**

**- File name:** Medical students’ survey.

**- Title of data:** Developing competencies to deal with intimate partner violence in Mozambican medical students.

**- Description of data:** Background information and self-efficacy scale**.**

**Section I: Background information**

| 1. Which best describes your gender identity? | Woman _____  Man _____  Transgender woman ____  Transgender man ____  Genderqueer or gender non-conforming ____ Questioning ____  Other (please, specify) ___________  Decline to state ____ |
| --- | --- |
| 1. Do you consider yourself to be: 2. Age | ­­­­­­­­Heterosexual or straight ____  Men Having Sex With men or lesbian ____  Bisexual ____  Asexual ____  Questioning ____  Not listed ____  Decline to state ____  ____ |
| 1. Marital status | Married ____ Single ____  Cohabiting ____ Divorced _____  Widow ______ |
| 1. Year in the medical curriculum | _____ |
| 1. Number of IPV survivors assisted | __________ |
| 1. Own IPV experience | Yes _______ No ______ |
| 1. Having witnessed IPV in own family | Yes _______ No ______ |

**Section II: Your medical training about Intimate Partner Violence**

The way you respond is easy. You give us a number between 0 and 100. This number refers to the % of Bachelor of Medicine in which Intimate Partner Violence has been dealt with. Fill out a % next to each element.

| **0** | 10 | 20 | 30 | 40 | **50** | 60 | 70 | 80 | 90 | **100** |
| --- | --- | --- | --- | --- | --- | --- | --- | --- | --- | --- |
| ⮙ |  |  |  |  | ⮙ | ⮙ |  |  |  | ⮙ |
| I do not feel that I have studied this subject. |  |  |  |  | I feel that I have learned the basic knowledge of the subject or skill set so that I can recognize and remember (name and describe) the material. | I feel that I have learned this subject at an intermediate level so that I am able to analyse a situation and use skills / knowledge correctly in isolation. |  |  |  | I feel that I have come to an advanced understanding of this subject and am able to integrate and adapt this skill / knowledge with others in order to create a new idea / experience. |

**KNOWLEDGE**

| Being able to define intimate partner violence. | % |
| --- | --- |
| Understanding common presenting signs symptoms and ""stories"" of intimate partner violence of survivor and abusers. |  |
| Understanding predisposing and predicting characteristics of abusers and survivors. |  |
| Understanding the epidemiology of intimate partner violence as it relates to race, gender, and sexuality, and its relation to substance abuse. |  |
| Understanding barriers to an abused 'person' admitted that s/'he' abused and seeking help. |  |
| Understanding common barriers for a physician to identify a survivor of intimate partner violence. |  |
| Understanding the limits of confidentiality surrounding discussion of intimate partner violence. |  |
| Understanding the legal requirements regarding disclosure of intimate partner violence and victimization to police. |  |
| Understanding treatment options for abusers and their relative chance of success. |  |
| Understanding the various community resources useful in caring for the survivor of intimate partner violence. |  |
| Understanding the psychological skills necessary to treat a survivor of intimate partner violence such as empathy and concern for safety. |  |

**ATTITUDES**

| Follow the needs to screen all patients for the issue of intimate partner violence and feelings of being unsafe in their homes. | % |
| --- | --- |
| Appreciate the multi-disciplinary nature of caring of the survivor of intimate partner violence. |  |
| Appreciate the profound impact that intimate partner violence can have on adult survivor and children. |  |
| Overcome stereotypes surrounding intimate partner violence (e.g., ethnic minorities as ""classic"" abusers). |  |

**SKILLS**

| Being able to conduct a detailed history when intimate partner violence is suspected. | % |
| --- | --- |
| Being able to conduct a screening history for intimate partner violence (when not suspected). |  |
| Being able to develop a strategy for interacting with a local community when an IPV case is identified (such as police, social services). |  |
| Being able to properly document intimate partner violence cases. |  |
| Being able to interview IPV survivors in an empathic, non-judgmental manner. |  |
| Being able to conduct interviews with proper attention to body position and interactions with the IPV abuser. |  |
