# Supplementary material for: Improving medical students’ communication competencies to deal with intimate partner violence using clinical simulations in Mozambique
Source: BMC Med Educ. 2021 Feb 23;21:126. doi: 10.1186/s12909-021-02560-8 (PMC7901116; doi:10.1186/s12909-021-02560-8)
Supplement: Supplementary file 2 — Additional file 2. [file 12909_2021_2560_MOESM2_ESM.docx]

**Additional file 2**

**- File name:** Table 4.

**- Title of data:** Case scenarios.

**- Description of data:** case scenarios used for simulation activities.

Table 4. Case scenarios

| **Scenario #** | **Content** |
| --- | --- |
| **Scenario 0** | This baseline scenario involved students in a generic consultation setting in which different script elements were demonstrated. The first author of this article adopted the role of physician and one participant took the other role. In addition to an introduction to the script, this scenario also allowed students to get acquainted with the clinical simulation format and in particular, the role-playing part. |
| **Scenario 1** | a female married survivor accompanied by her mother. This scenario aimed at understanding privacy issues in dealing with IPV survivors, recognizing abuse, and validating patient emotions. This was developed taking into considering that the female married survivor was not willing to disclose abuse to her mother. |
| **Scenario 2** | a woman (survivor) with husband (assailant or alleged assailant) – they are a couple. This scenario was expected to help identify a survivor of IPV and watch and interpret non-verbal cues that indicate anxiety or distress, to evaluate the related health problems and safety needs of the patient, and to develop a safety plan. |
| **Scenario 3** | a man (survivor) with a man (assailant or alleged assailant) – man having sex with a man (MSM) in a romantic relationship. The focus of this scenario was to understand IPV epidemiology and practice attitudes to overcome personal bias (diversity, sexual orientation biases). We also expected to help in using referral/linkage of the patient to community resources and to develop a safety plan. |
